# Orientational ordering and assembly of silica–nickel Janus particles in a magnetic field

**DOI:** 10.1107/S205225252301000X

**Published:** 2024-01-01

**Authors:** Gouranga Manna, Thomas Zinn, Lewis Sharpnack, Theyencheri Narayanan

**Affiliations:** a ESRF – The European Synchrotron, 38043 Grenoble, France; Lund University, Sweden; Keele University, United Kingdom

**Keywords:** nanoscience, small-angle X-ray scattering, nanostructure, magnetic Janus particles, magnetic field induced orientations, X-ray photon correlation spectroscopy, anisotropic scattering, particle dynamics

## Abstract

The orientation ordering and assembly behavior of silica–nickel Janus particles in a static external magnetic field were probed by ultra small-angle X-ray scattering (USAXS). Even in a weak applied field, the net magnetic moments of the individual particles aligned in the direction of the field, as indicated by the anisotropy in the recorded USAXS patterns. X-ray photon correlation spectroscopy (XPCS) measurements on these suspensions revealed that the corresponding particle dynamics are primarily Brownian diffusion [Zinn, Sharpnack & Narayanan (2023 ▸). *Soft Matter*, **19**, 2311–2318]. At higher fields, the magnetic forces led to chain-like configurations of particles, as indicated by an additional feature in the USAXS pattern. A theoretical framework is provided for the quantitative interpretation of the observed anisotropic scattering diagrams and the corresponding degree of orientation. No anisotropy was detected when the magnetic field was applied along the beam direction, which is also replicated by the model. The method presented here could be useful for the interpretation of oriented scattering patterns from a wide variety of particulate systems. The combination of USAXS and XPCS is a powerful approach for investigating asymmetric colloidal particles in external fields.

## Abstract 2

The alignment and self-assembly behavior of silica–nickel Janus particles in an external magnetic field were probed by ultra small-angle X-ray scattering. A theoretical framework is provided for the quantitative interpretation of the observed anisotropic scattering features.

## 1. Introduction

Magnetic colloidal particles dispersed in a solvent display a variety of distinct responsive behavior in the presence of an external magnetic field (Safran, 2003 ▸; Lemaire *et al.*, 2005 ▸; Faraudo *et al.*, 2016 ▸). As the interactions can easily be tuned by the applied magnetic field, these nanomaterials are highly appealing for many practical applications. For example, magnetic separation (Kelland, 1998 ▸), magnetically controlled colloidal crystallization (Pal *et al.*, 2015 ▸; Taheri *et al.*, 2015 ▸; Li *et al.*, 2021 ▸) or biomedical applications like magnetically driven targeted delivery (Vuong *et al.*, 2012 ▸; Lucena *et al.*, 2021 ▸) are a few to mention. In a magnetic field, these particles often assemble into different anisotropic structures, such as linear chains or chain-like zigzag strands or densely packed double chains (Safran, 2003 ▸; Wang *et al.*, 2013 ▸; Rikken *et al.*, 2014 ▸). Advances in particle synthesis now enable one to realize different shapes or magnetic properties of the particles (Poggi & Gohy, 2017 ▸; Su *et al.*, 2019 ▸). These magnetic colloids possess either shape or magnetic anisotropy or both and typically sizes range from 20 nm to 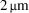 .

The equilibrium static structures formed by these particles under the influence of an external field have been well addressed (De Gennes & Pincus, 1970 ▸; Cerdà *et al.*, 2010 ▸; Hernández-Rojas *et al.*, 2016 ▸; Faraudo *et al.*, 2016 ▸; Ku *et al.*, 2016 ▸). Depending on the size range of these particles, the theoretical predictions may be verified by optical microscopy (Smoukov *et al.*, 2009 ▸; Swan *et al.*, 2014 ▸) or scattering methods such as small-angle X-ray and neutron scattering (SAXS and SANS, respectively) (Paula, 2019 ▸; Kamal *et al.*, 2020 ▸; Wiedenmann *et al.*, 2006 ▸; Barrett *et al.*, 2011 ▸; Petrenko *et al.*, 2018 ▸; Nandakumaran *et al.*, 2021 ▸) or by computer simulations (Lago *et al.*, 2003 ▸; Peng *et al.*, 2009 ▸; Kantorovich *et al.*, 2011 ▸). Traditionally, light scattering has been used to probe the dynamics of these systems (Donselaar & Philipse, 1999 ▸; Martínez Pedrero *et al.*, 2006 ▸). Over recent decades, new techniques such as optical particle tracking (Kaiser *et al.*, 2017 ▸), dynamic differential microscopy (Pal *et al.*, 2020 ▸) and X-ray photon correlation spectroscopy (XPCS) (Lal *et al.*, 2001 ▸; Wagner *et al.*, 2005 ▸, 2013 ▸; Wandersman *et al.*, 2009 ▸; Pal *et al.*, 2021 ▸) have emerged for investigating the dynamics of these systems. Nevertheless, a detailed understanding of the self-assembly behavior and underlying dynamics remains incomplete. In this work, spherical Janus colloids with a magnetic hemispherical cap have been investigated. Janus particles (JPs) with a magnetic anisotropy have received interest in recent years from the point of view of magnetically guided propulsion (Huhnstock *et al.*, 2021 ▸).

The interaction potential, *U*(*r*, Θ), between magnetic particles is anisotropic and depends on the angle, Θ, between the line joining the centers of the two dipoles and the magnetic field, **B** (Faraudo *et al.*, 2016 ▸; Ku *et al.*, 2016 ▸). When the magnetic moment is along the field direction, *U*(*r*, Θ) systematically evolves from repulsive to attractive as Θ is decreased from 90 to 0° (Faraudo *et al.*, 2016 ▸). By considering the induced magnetization and other orientations, *U*(*r*, Θ) becomes even more complex (Ku *et al.*, 2016 ▸). Nevertheless, the maximum attraction is obtained when two particles are in contact with their magnetic dipoles aligned in the direction of **B** and the maximum repulsion is attained for the perpendicular configuration. These direction-specific interactions are at the origin of anisotropic structures formed when a magnetic field is applied (Faraudo *et al.*, 2016 ▸).

In the present work, the microstructure and orientation behavior of magnetic JPs at the interparticle level were elucidated using SAXS and ultra SAXS (USAXS). The main emphasis was to formulate a theoretical framework for the quantitative modeling of the observed orientation behavior. To complement the anisotropic structural information, the corresponding particle dynamics were probed by XPCS (Zinn *et al.*, 2023 ▸). The colloidal system consisted of spherical silica particles with a hemispherical nickel cap suspended in water. In order to minimize magnetic dipole–dipole interactions between the particles prior to applying the magnetic field, relatively dilute samples with colloid volume fractions (ϕ) ranging from 10^−5^ to 10^−4^ were used. In these dilute conditions, the mean separation between two particles was relatively large and the suspension remained stable for several days (Semeraro *et al.*, 2018 ▸). At low values of |**B**|, the particles preferably aligned along the field while the dynamics still displayed diffusive behavior. In stronger fields, magnetic interactions led to chain-like assemblies (Zinn *et al.*, 2023 ▸), which can be simulated by the proposed framework. The presented approach will be applicable to the alignment behavior of a variety of anisotropic particles in external fields such as electric or magnetic, in shear flow and in confinement.

## 2. Materials and methods

Samples and X-ray scattering methods were essentially the same as in the previous publication (Zinn *et al.*, 2023 ▸) and are therefore only briefly described for the sake of completeness.

### 2.1. Materials

JPs were fabricated from spherical silica colloids of radius *R* = 220 nm onto which a hemispherical nickel cap (magnetic susceptibility, χ ≃ 600) of thickness *d* ≃ 40 nm was sputtered (Semeraro *et al.*, 2018 ▸). The coated particles were extracted and fractionated as described before (Semeraro *et al.*, 2018 ▸). The purified JPs were suspended in milli-Q water and filled in quartz glass capillaries of diameter 1 mm. A |**B**| from 1.0 mT up to 1.0 T was applied using a permanent magnet setup in the Halbach arrangement (Zinn *et al.*, 2023 ▸).

### 2.2. X-ray scattering

USAXS measurements were performed simultaneously with XPCS on the TRUSAXS beamline (ID02) at the European Synchrotron Radiation Facility (ESRF) in Grenoble (France) (Narayanan *et al.*, 2018 ▸). The incident X-ray energy was 12.46 keV, corresponding to a wavelength of λ = 0.0995 nm. Most of the measurements were carried out at a sample-to-detector distance of 30.7 m covering a *q* range from 0.002 to 0.1 nm^−1^, where *q* is the magnitude of the scattering vector **q**, given by 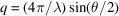 with θ as the scattering angle. The full width at half-maximum *q* resolution of the setup was of the order of 2.5 × 10^−4^ nm^−1^. Additional measurements were carried out at a sample-to-detector distance of 1.5 m to record the high *q* part of the scattering form factor of JPs up to 2.0 nm^−1^. Two-dimensional SAXS/USAXS patterns were acquired using the Eiger 500 K detector (Zinn *et al.*, 2018 ▸) with a pixel size of 75 µm. One-dimensional SAXS/USAXS intensity profiles denoted by *I*(*q*) were obtained after applying different detector corrections and normalization to an absolute scale (Narayanan *et al.*, 2018 ▸), and averaging over the specified azimuthal range. Further processing of the 2D scattering patterns was performed using the *SAXSutilities* software (Sztucki, 2021 ▸).

For the modeling, the orientation-averaged 1D USAXS profiles were calculated in Python (Release 3.6) and the 2D patterns were generated using *Mathematica* (Version 10.4) (Wolfram, 2014 ▸). The drawings were realized using the open-source software *Inkscape* (Inkscape Contributors, 2022 ▸). Details of XPCS data treatment and analysis were presented in the previous publication (Zinn *et al.*, 2023 ▸).

## 3. Theoretical framework

### 3.1. Calculation of the scattering function of oriented Janus particles

**Figure 1:**
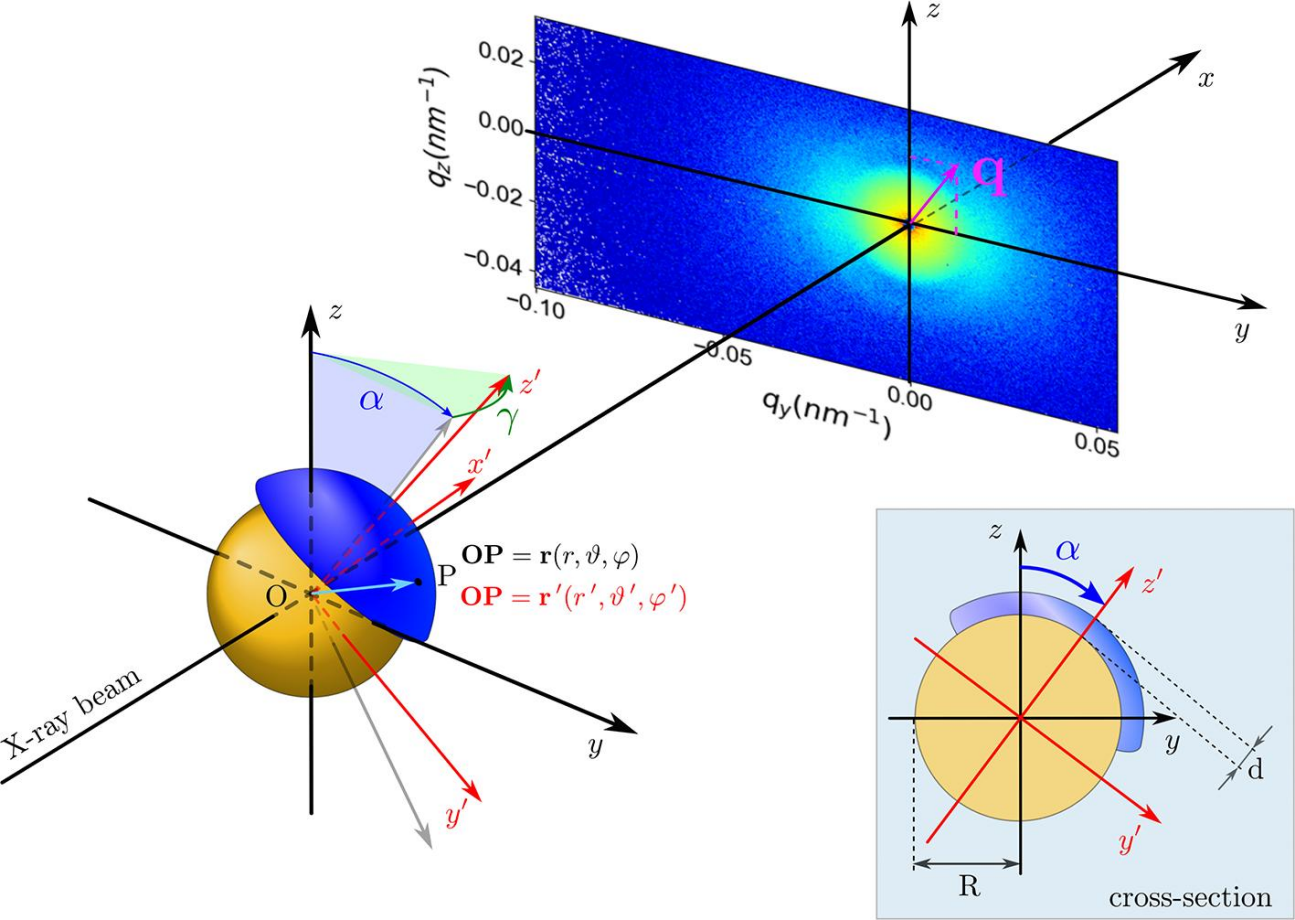
A schematic representation of a JP and the scattering geometry involved in the calculation. The axis of the particle is rotated with respect to the *x* and *z* axes.

While there have been several attempts to model the orientation-averaged scattering function of JPs (Kaya, 2002 ▸; Fütterer *et al.*, 2004 ▸; Semeraro *et al.*, 2018 ▸; Anitas, 2020*a* ▸,*b* ▸), there is not a complete description of oriented or partially oriented JPs available in the literature yet. Oriented SAXS patterns of anisotropic objects can be computed via a 2D indirect Fourier transform method (Fritz-Popovski, 2013 ▸) or oriented pair distribution functions (Alves *et al.*, 2017 ▸) and reverse Monte Carlo simulations (Nandakumaran *et al.*, 2021 ▸). Here, a different attempt is made to simulate JPs that orient due to the surface anisotropy in an external magnetic field, **B**. In principle, the results are valid for any type of orientation such as shear flow or an external electric field. Based on experimental observation (Zinn *et al.*, 2023 ▸) and theoretical expectation (Ku *et al.*, 2016 ▸), JPs are considered to freely move but rotate with their symmetry axis pointing in the direction of **B**. Each JP is composed of two domains (the core and the cap) with different scattering-length densities 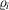 , as schematically depicted in Fig. 1 ▸, and their scattering contrast in a medium is given by 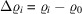 , with 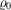 being the scattering-length density of the suspending medium (here, water). In general, the (anisotropic) scattered intensity *I*(**q**) of JPs [*I*_JP_(**q**)] is given by 

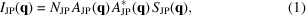

where *N*_JP_ is the number density of JPs in the scattering volume, *A*_JP_(**q**) is the scattering amplitude of an individual JP and *S*_JP_(**q**) is the structure factor of interaction between JPs. For the very dilute samples investigated in this work (ϕ ≤ 10^−4^), in weak magnetic fields *S*_JP_(**q**) ≃ 1 and can be neglected. The scattering amplitude of a particle of volume *V* and scattering contrast 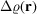 can be written as (Guinier, 1994 ▸) 

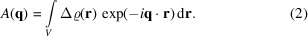

The scattering amplitude of the JP is given by the sum of a solid sphere of radius *R* and scattering-length density 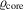 and a hemispherical cap with scattering-length density 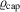 and thickness *d* (Kaya, 2002 ▸), *i.e.*


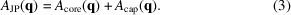

The scattering amplitude of the core is given by the usual expression for a solid sphere (Guinier, 1994 ▸) with a continuous distribution of 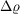 , see Appendix *A* [App appa]. To calculate the 2D scattering pattern in the detector plane (assuming that the *x* direction is along the X-ray beam), the intensity variations along *q*_*y*_ and *q*_*z*_ are required, *i.e.* **q** = (0, *q*_*y*_, *q*_*z*_). Hence, with 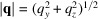 and a constant 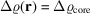 , the scattering amplitude of the core becomes 

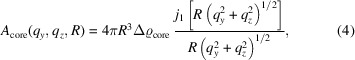

where *j*_1_(·) is the first-order spherical Bessel function (Abramowitz & Stegun, 1964 ▸). In the most general case, the rotation of the particle needs to be incorporated into the model to describe different orientations. For the sake of simplicity, the particle reference frame (*x*′, *y*′, *z*′) is rotated around the common *x* axis by an angle α followed by a second rotation around the *z* axis by an angle γ with respect to the laboratory frame (*x*, *y*, *z*), as schematically shown in Fig. 1 ▸. This rotational transformation of the position vector **r**′ into **r** is given by the transformation matrix *R*_*z*,*x*_(γ, α) = *R*_*z*_(γ) · *R*_*x*_(α), see Appendix *B* [App appb]. Under the assumption of an initial alignment perpendicular to the beam direction, which is the *x*-coordinate axis, the angle 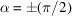 . By considering that **q** = (0, *q*_*y*_, *q*_*z*_) is in the *y*–*z* plane, then the phase term *i* **q** · **r** in equation (2) can be calculated using spherical coordinates of 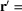 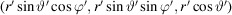 : 

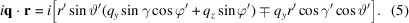

Following equation (2), the scattering amplitude for the cap is given by 

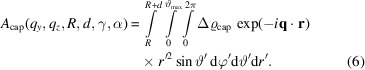

As shown in Appendix *C* [App appc], *A*_cap_ can be analytically written as 

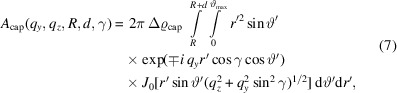

where *J*_0_(·) is the zeroth-order Bessel function of the first kind (Abramowitz & Stegun, 1964 ▸). The integration in equation (7) cannot be performed analytically but it can be evaluated numerically. For this purpose, the double integral is converted into a summation. The simplest way to calculate a definite integral numerically is to use the Riemann sum method. Additionally, for the integration over *r*′, a variation of the cap thickness *d* is introduced according to 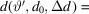 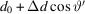 . The Riemann sums are given in Appendix *D* [App appd]. The full numerical expression of the scattering amplitude *A*_cap_ for the cap becomes 

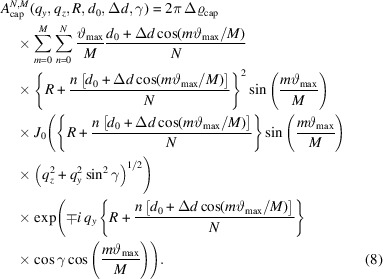

Using equations (4) and (8), the total scattering amplitude *A*_JP_ is given by 

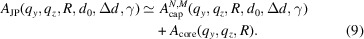

Here *A*_JP_(*q*_*y*_, *q*_*z*_, *R*, *d*_0_, Δ*d*, γ) is a complex function that can be written as

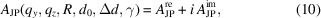

with 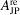 and 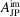 being the real and imaginary parts of *A*_JP_(*q*_*y*_, *q*_*z*_, *R*, *d*_0_, Δ*d*, γ), respectively. Note that 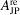 and 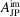 are real functions of *q*_*y*_, *q*_*z*_, *R*, *d*_0_, Δ*d* and γ. For simplification, the individual arguments of the functions 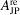 and 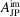 are not explicitly written any further in the following.

For the analysis of real particle suspensions, *I*_JP_(**q**) needs to be further convoluted by a size distribution *f*(*r*) (polydispersity). Without loss of generality, here a Gaussian size distribution with mean radius *R* and standard deviation σ_*R*_ was used: 

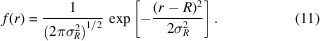

Therefore, the polydisperse scattered intensity is given by 

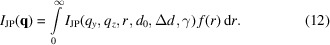

For randomly oriented JPs, the isotropic intensity *I*_JP_(*q*) is obtained by the orientational average of equation (12) over the solid angle Ω using equation (9): 

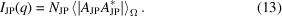




### 3.2. Scattering function of oriented doublets

**Figure 2:**
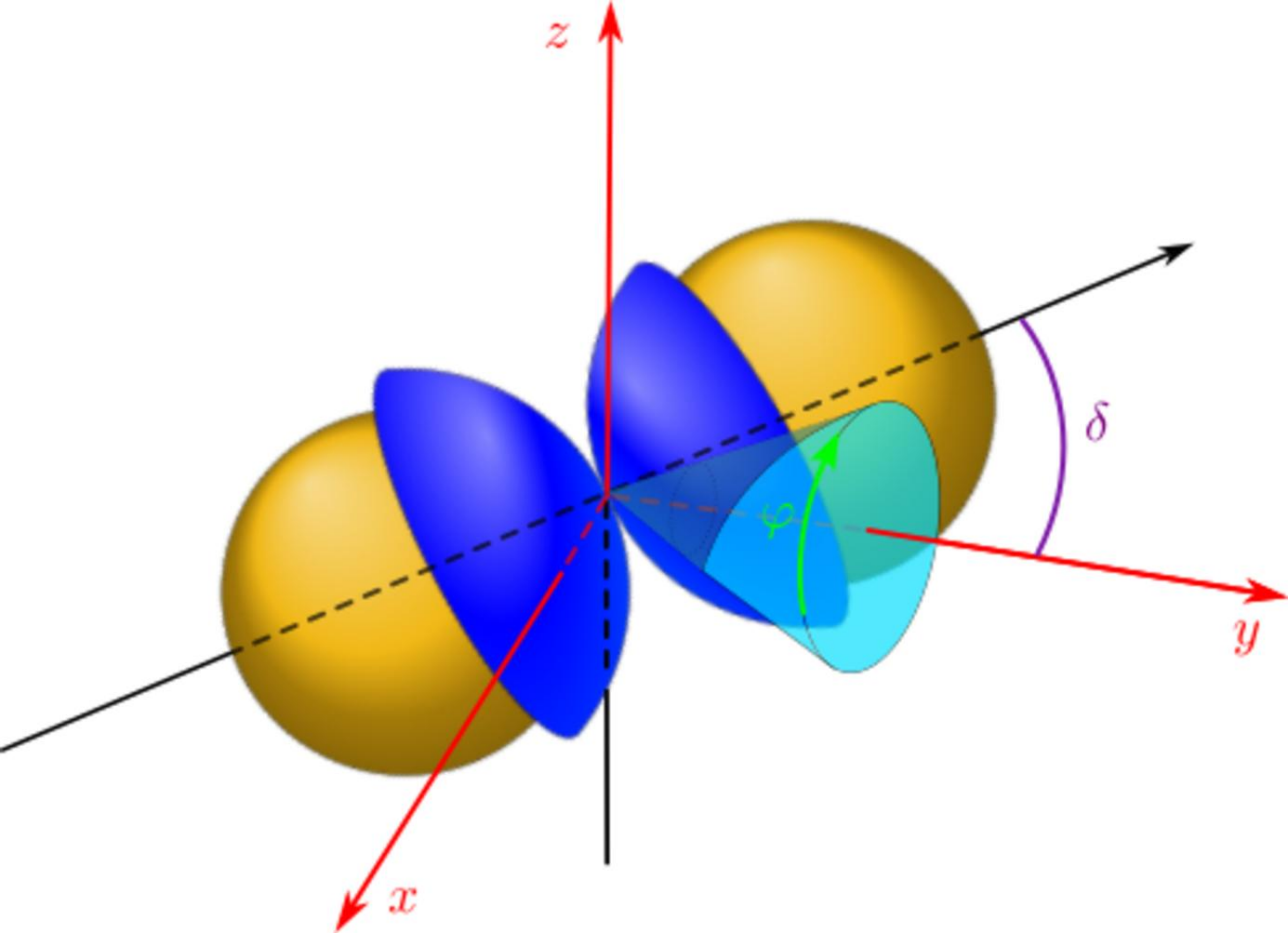
A schematic representation of two JPs in a doublet arrangement.

Experimentally, a butterfly-like scattering pattern is observed at higher magnetic fields (Zinn *et al.*, 2023 ▸). As a first step towards describing this scattering feature, two JPs with their caps facing each other are considered, as schematically depicted in Fig. 2 ▸. The scattering amplitude of such a doublet can be obtained by 

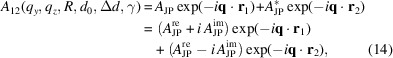

with **r**_1,2_ denoting the position vector of the two JPs. Using the property

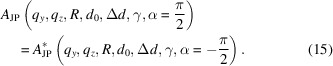

The corresponding scattered intensity of a doublet is given by 

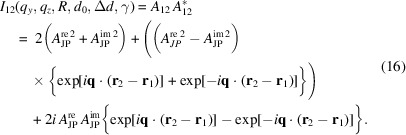

For the sake of simplicity, it is assumed that JPs are oriented along the magnetic field. However, the line connecting the two JP centers may be slightly tilted by an angle δ with respect to the magnetic field. The doublet of JPs is oriented in the *x*–*z* plane with possible orientation around the magnetic field direction subtending a solid angle around the *y* axis. To consider all these different orientations of **r**_1_ and **r**_2_, the rotation matrix *R*_*y*,*y*_(φ) is introduced such that an orthogonal rotation by an angle φ ∈ [0, 2π] around the *y* axis. By replacing the expression, the scattered intensity *I*_12_(*q*_*y*_, *q*_*z*_, *R*, *d*_0_, Δ*d*, γ) (details are given in Appendix *E* [App appe]) can be written as 

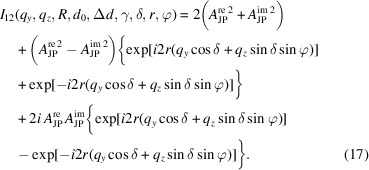

Averaging over all φ leads to a simplification of equation (17): 

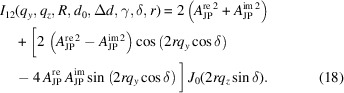

Following the same procedure, *I*_12_(*q*_*y*_, *q*_*z*_, *R*, *d*_0_, Δ*d*, γ, δ, *r*) for the case of a magnetic field parallel to the X-ray beam (*x* axis) can be written as 

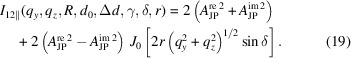

Corresponding polydisperse scattered intensities are obtained by the convolution of equations (18) and (19) with *f*(*r*).

### 3.3. Structure factor of chain-like configurations

**Figure 3:**
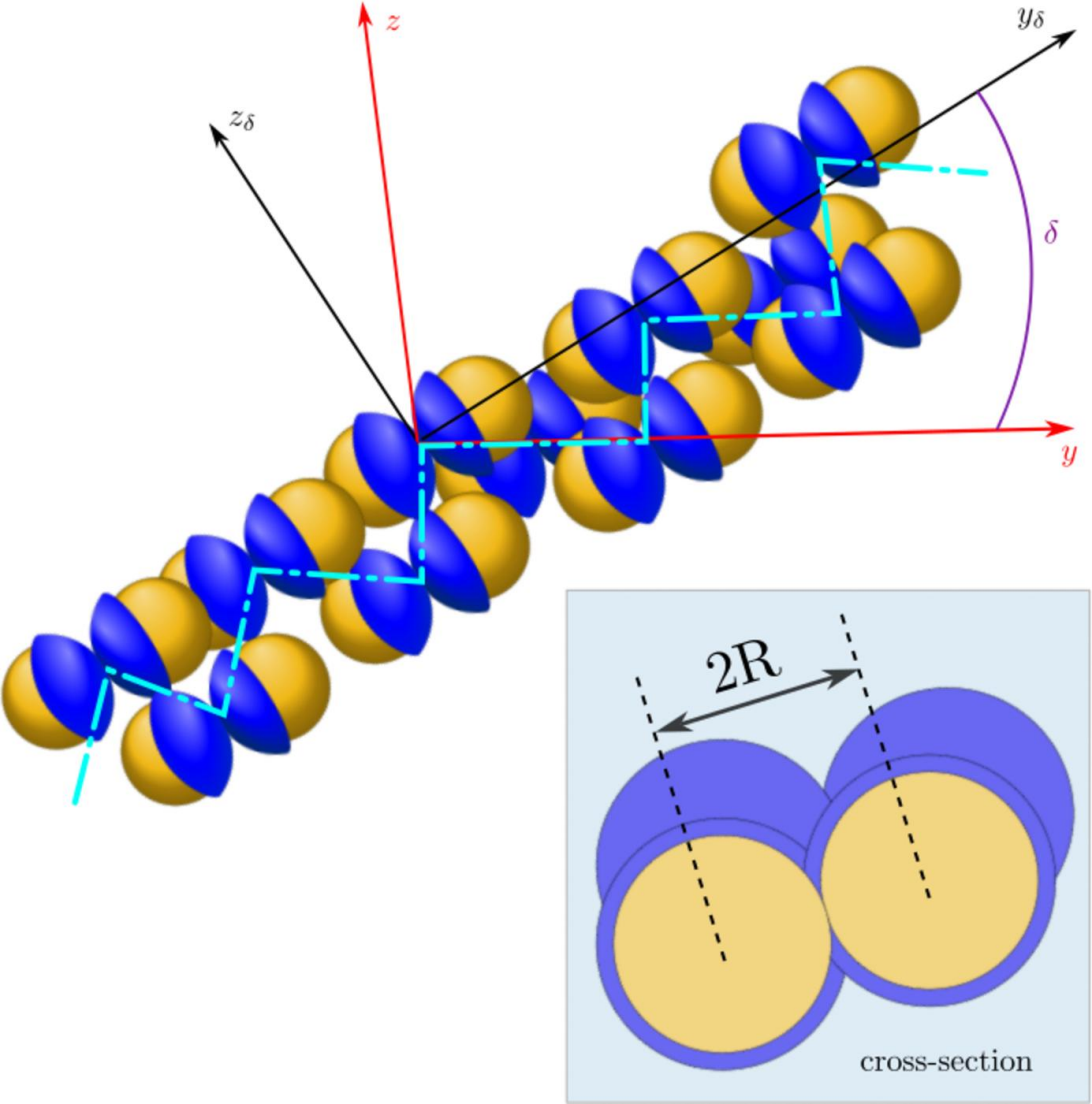
A schematic representation of JPs in a chain-like arrangement. The zigzag-arrangement of JP doublets are indicated by the light blue dash-dotted line.

The magnetic dipole moment of the doublets formed in stronger fields further interacts with the dipole moments of other doublets or isolated singlets. In this case, the most favorable morphology will be oriented chain-like aggregates, which maximizes the magnetic interactions. In the doublets, the nickel caps are in between two JPs and the next particle must lie in such a way that its cap is closest to the doublet caps, as schematically depicted in Fig. 3 ▸. This leads to an inclined chain or zigzag arrangement. To describe the scattering contribution due to zigzag chains, the 2D paracrystal structure factor may be considered (Guinier, 1994 ▸). Based on a similar strategy used to calculate the scattered intensity of oriented doublets, the structure factor of an oriented chain can be calculated. If **r**_*n*_ is the position of the *n*th doublet with respect to the first particle, then the structure factor *S*_ch_(*q*_*y*_, *q*_*z*_, *r*, σ_*y*_, σ_*z*_) for a finite chain can be expressed as (Eads & Millane, 2001 ▸) 

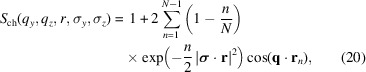

where 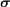 = (0, σ_*y*_, σ_*z*_), and the components 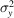 and 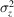 are the variances of 〈|**r**_*n*+1_ − **r**_*n*_|〉 along the *y* and *z* axes, respectively. *N* denotes the number of doublets in the chain structure. Assuming that the chain axis is tilted by an angle δ with respect to **B**, then equation (20) is given by

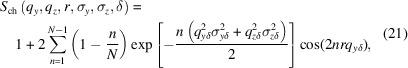

with the following parameters 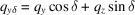 , 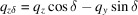 and 

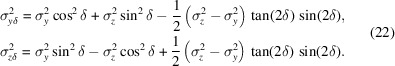

Finally, the chain structure is allowed to have a restricted orientational freedom by a maximum angle δ_max_ = ±30°. The resulting orientationally averaged structure factor is numerically given by 

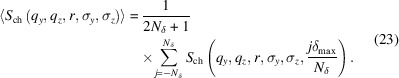

For clarity, δ is the solid angle subtended around **B** that is applied along the *y* direction. Therefore, the projections *q*_*y*,*z*_ and σ_*y*,*z*_ are asymmetric in the *y*–*z* plane. However, for the case where **B** is applied along the X-ray beam direction (*x* axis), the projections *q*_*y*,*z*_ and σ_*y*,*z*_ become symmetric in the *y*–*z* plane and equation (20) reduces to

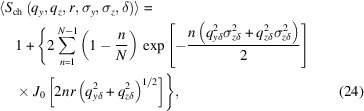

with *q*_*y*δ_ = *q*_*y*_, 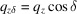 and 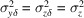 .

In the calculations, the orientational averaging was performed on the total *I*_JP_(**q**) rather than *S*_ch_(**q**).

## 4. Results and discussion

This section presents a comparison of the normalized USAXS patterns with the model presented above. XPCS results and analysis were reported in the previous publication (Zinn *et al.*, 2023 ▸) and are therefore not repeated here. Nevertheless, for the sake of completeness, Fig. 1 of the supporting information depicts the main dynamic features of this suspension of JPs.

### 4.1. Application to randomly oriented Janus particles

**Figure 4:**
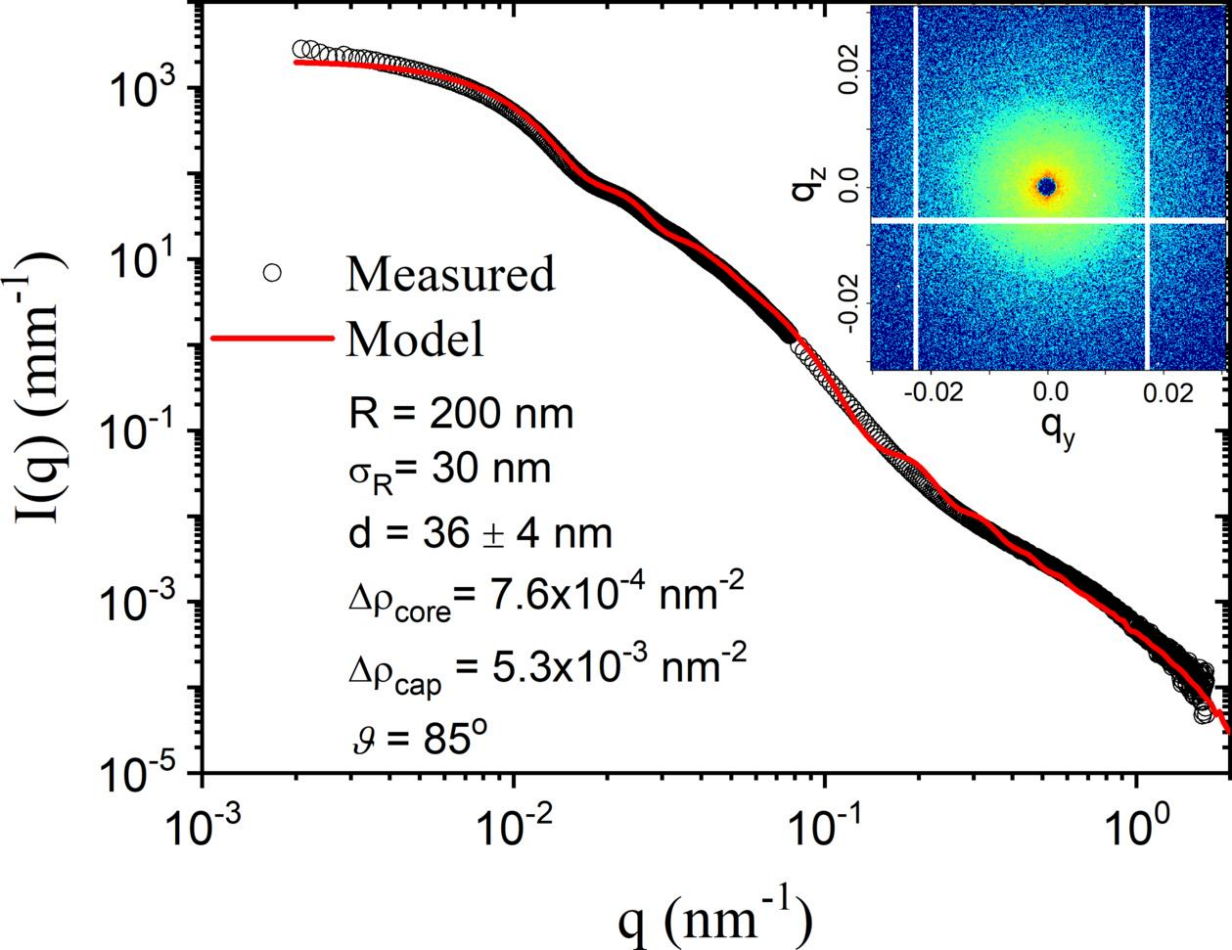
A normalized SAXS/USAXS profile from a dilute suspension of JPs (ϕ ≃ 3 × 10^−5^). The parameters for the best-fit model using equation (13) are listed in the legend. The different symbols are defined in the main text. The inset depicts an isotropic scattering pattern in the absence of a magnetic field, |**B**| = 0.

As a first test of the presented model, Fig. 4 ▸ shows the 1D scattering profile of JPs measured using a dilute suspension (ϕ ≃ 3 × 10^−5^) over an extended *q*-range without an applied magnetic field (*i.e.* |**B**| = 0). The background scattering contributions were accurately subtracted after the normalization and azimuthal averaging of the isotropic 2D scattering patterns. Equation (13) with a Gaussian size distribution adequately describes the data, with the parameters listed in the legend of Fig. 4 ▸. To obtain a satisfactory description at high *q*, the cap thickness *d* was varied from 22 nm at the edges to 50 nm at the middle of the cap. The median of 36 nm is close to the value of the nickel cap thickness reported for these particles (Zinn *et al.*, 2020 ▸, 2023 ▸) using a constant *d* model. However, the high *q* part of the scattering profile was not analyzed in those works. In addition, the presented model is also able to estimate the surface coverage of the nickel cap. The slight deviation at the low *q* region can be related to the presence of some doublets in the suspension (Semeraro *et al.*, 2018 ▸). The estimated variations of thickness and surface coverage are attributed to the fabrication procedure via sputtering onto a fixed target. The dispersity in *d* is not considered and the fine feature of the model curve around *q* of 0.2 nm^−1^ is attributed to this exclusion.

### 4.2. Orientation behavior of Janus particles in a magnetic field

**Figure 5:**
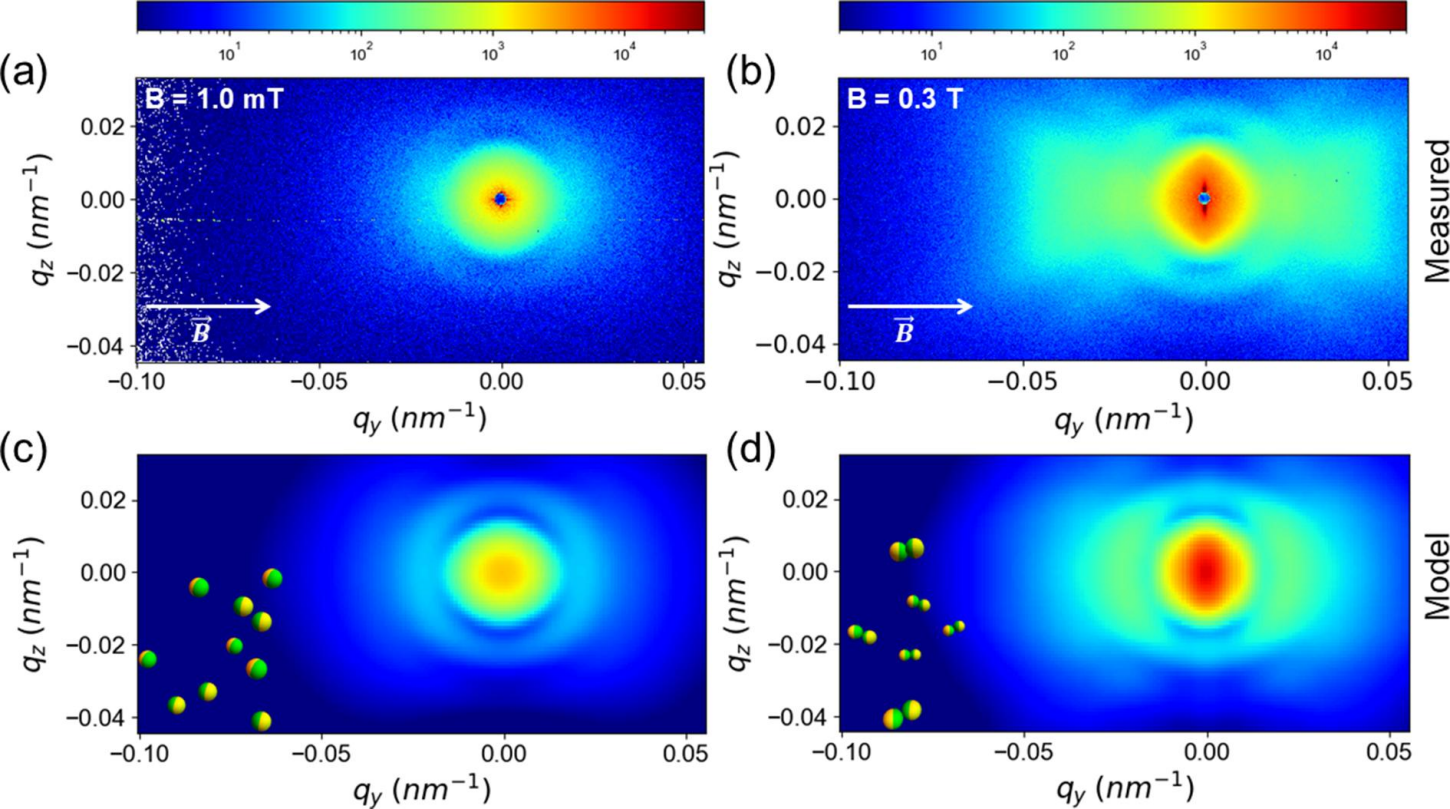
Representative orientation behavior of JPs at two different magnetic fields along the *y* axis: (*a*) |**B**| = 1.0 mT and (*b*) |**B**| = 0.3 T. Corresponding simulated 2D patterns (*c*) and (*d*) were generated by equations (12) and (18), respectively, with the same structural parameters as in Fig. 4 ▸ for (*c*) γ = ±45°, and (*d*) γ = ±30° and δ = 30°. Insets in (*c*) and (*d*) depict the orientation/association of JPs schematically.

The main goal of this study is to offer a quantitative description of the alignment of JPs observed in an applied magnetic field, **B**. The top part of Fig. 5 ▸ displays the typical USAXS patterns from a suspension of JPs (ϕ ≃ 10^−4^) at two different |**B**| values applied along the *y* axis. A similar behavior can be found even for lower ϕ and other magnitudes of **B** (Zinn *et al.*, 2023 ▸). The scattering pattern in the absence of an applied field is fully isotropic (*c.f.* Fig. 4 ▸) but a clear anisotropy is visible for the lowest applied field of 1.0 mT (*i.e.* the magnetic poles fully opened). This anisotropy even in a weak field and at low ϕ can be attributed to strong magnetic interactions. The bottom part of Fig. 5 ▸ presents simulated 2D USAXS patterns using the model derived above for the same structural parameters as in Fig. 4 ▸. Pattern (*c*) was generated using equation (12) and γ = ±45° for singlets. While pattern (*d*) was generated using equation (18), 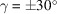 and 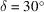 for doublets with caps facing each other (Fig. 2 ▸). These parameters imply that the orientation is only partial. The anisotropy became more pronounced with increasing |**B**| beyond 0.3 T. The corresponding analysis, which involved the structure factor of chain-like zigzag arrangements of JPs, will be presented in the next subsection. The central anisotropy along the vertical direction (*i.e.* *q*_*z*_) in Figs. 5 ▸(*b*) and 5 ▸(*d*) already indicates an onset of elongated structures along the field direction, while the lobes of the anisotropic patterns at higher *q* values correspond to the correlation between the magnetic caps when they come face-to-face in an aligned doublet or cluster. A key finding of this analysis is in fact the identification of the four lobes or butterfly pattern from the face-to-face configuration of the nickel caps.

**Figure 6:**
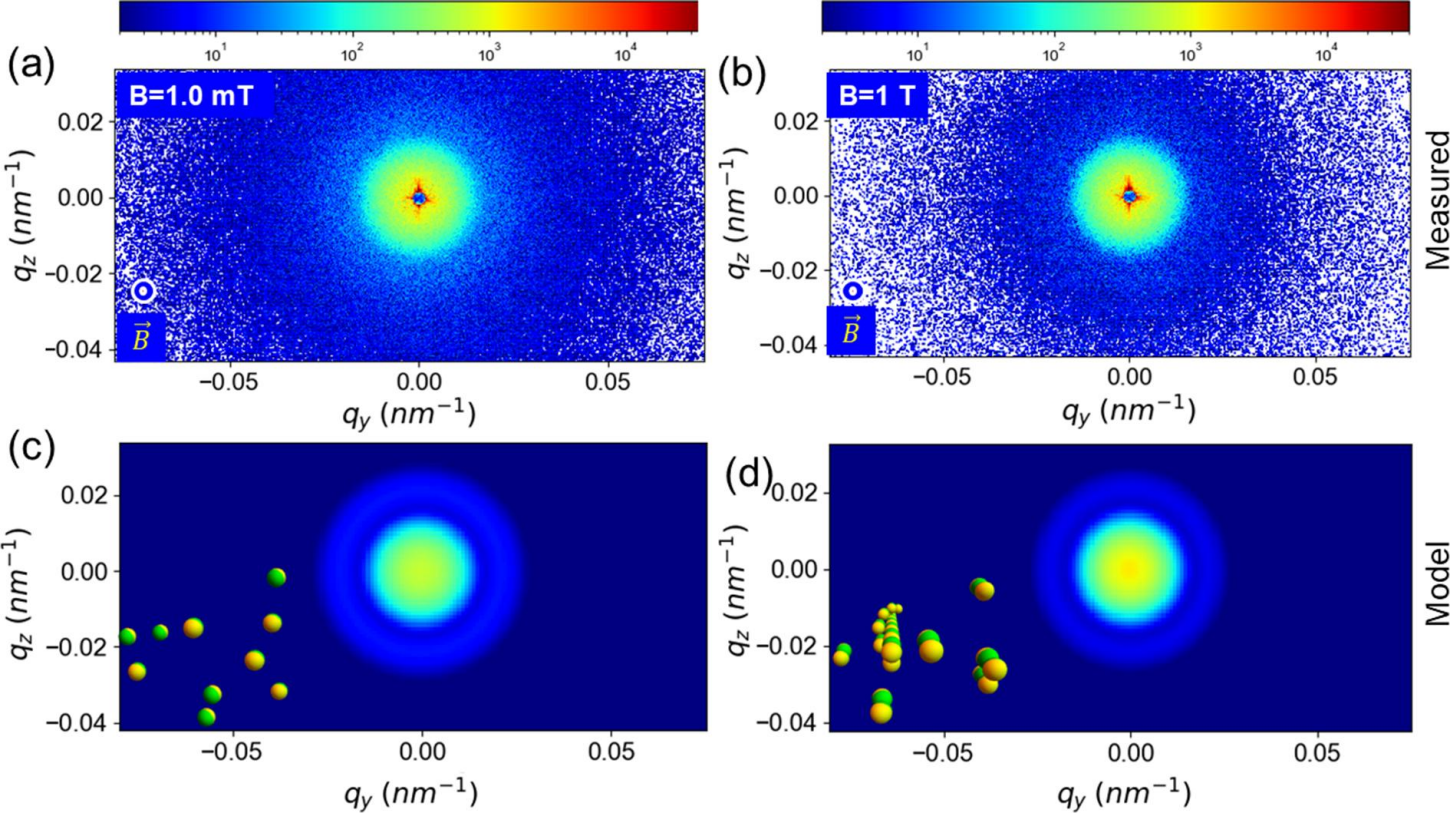
Representative isotropic 2D USAXS patterns from JPs at two different values of **B** applied along the X-ray beam direction (*x* axis): (*a*) |**B**| = 1.0 mT and (*b*) |**B**| = 1 T. Corresponding simulated 2D patterns were generated using the same structural parameters as in Fig. 4 ▸ by (*c*) equation (12) with γ = ±45° (single), and by (*d*) equation (19) with γ = ±30° and δ = 30° (doublets). Insets in (*c*) and (*d*) depict the orientation/association of JPs schematically.

In contrast, when the magnetic field was applied along the *x* axis, *i.e.* parallel to the X-ray beam, the scattering pattern remained fully isotropic over the range of **B** probed. Fig. 6 ▸ depicts two representative USAXS patterns: (*a*) |**B**| = 1.0 mT and (*b*) |**B**| = 1 T. Except for an increase of intensity at the lower *q* region, no striking feature can be discerned. An analogous behavior is replicated by the model, as indicated by the simulated patterns in Figs. 6 ▸(*c*) and 6 ▸(*d*), for the same structural parameters as in Fig. 4 ▸, with (*c*) γ = ±45° for singlets and (*d*) γ = ±30° and δ = 30° for doublets. This observation confirms that the magnetic moments of the nickel caps predominantly point in the direction of the particle axis of symmetry (Steinbach *et al.*, 2016 ▸).

**Figure 7:**
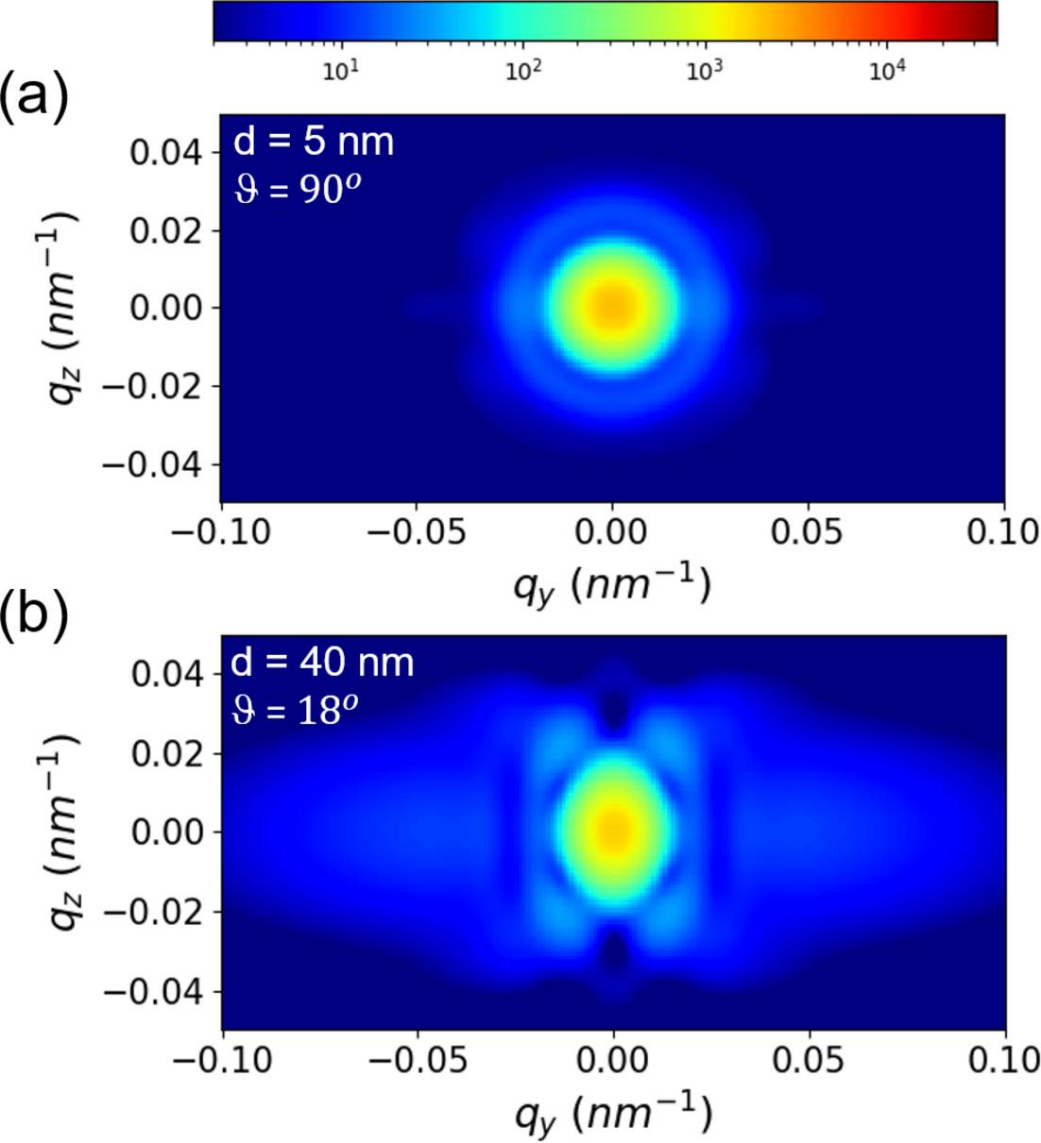
Simulated 2D USAXS patterns from JPs (singlets with γ = 0) at two different values of cap thickness (*d*) and surface coverage (ϑ) as indicated in the legends.

To complete the discussion on the orientation behavior of JPs, Fig. 7 ▸ shows the simulated orientation behavior using equation (12) (singlets with γ = 0) when the cap thickness and surface coverage are varied. The anisotropy is weaker with a thinner cap (Semeraro *et al.*, 2018 ▸), even with full hemispherical coverage. On the other hand, strong anisotropy is manifested with a thicker cap even if the surface coverage is partial. This suggests that a stronger magnetic force may be experienced by the latter, which will be a useful guide for the magnetic field induced self-assembly (Faraudo *et al.*, 2016 ▸).

### 4.3. Formation of chain-like assemblies in stronger magnetic fields

**Figure 8:**
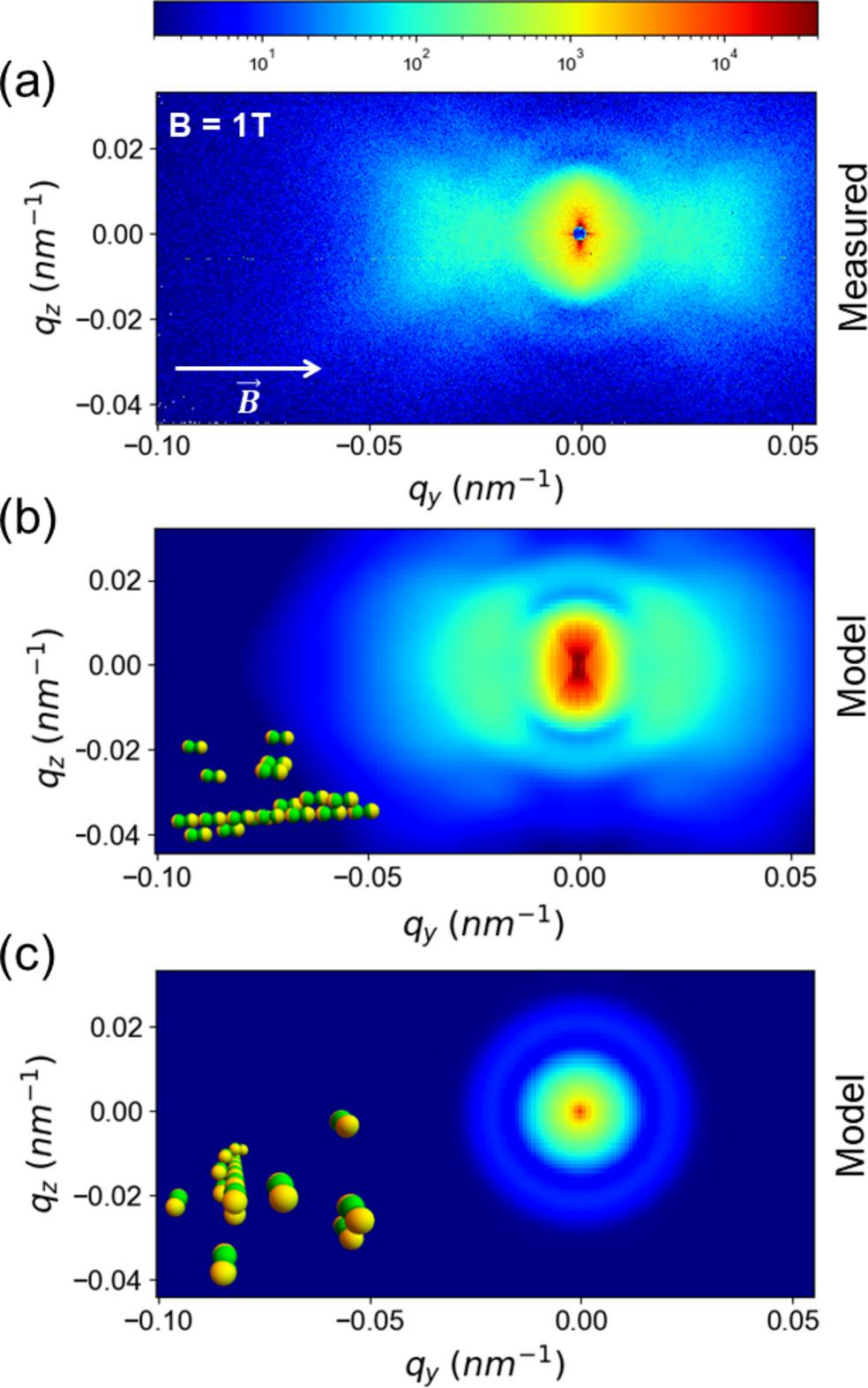
(*a*) A representative anisotropic 2D USAXS pattern from JPs for an applied **B** of 1 T perpendicular to the X-ray beam direction. Notice the central streak formed by the chain-like configurations of JPs. (*b*) Corresponding modeling in terms of equations (18) and (23) using the same structural parameters as in Fig. 4 ▸ for γ = ±30°, δ = ±30°, σ_*x*_ = 40 and σ_*y*_ = 60. The average number of JPs per chain was 20. The inset in (*b*) schematically depicts the orientation and twisted chain of the JPs. (*c*) For the same set of parameters, an isotropic pattern is obtained using equations (19) and (24) when the chains are oriented along the beam direction as sketched in the inset.

As the strength of the applied magnetic field is increased, not only are more doublets formed but also higher-order clusters. Fig. 8 ▸(*a*) presents representative patterns observed for a higher value of |**B**| = 1 T along the *y* axis (ϕ ≃ 10^−4^). The behavior is similar even up to 1.3 T, the highest value of |**B**| probed. The formation of the elongated structures as identified by the central streak became even more prominent. Under these conditions, XPCS intensity–intensity autocorrelation functions, *g*_2_(*q*, *t*), significantly deviated from a single exponential decay and turned to a compressed exponential form (see Fig. 1 of the supporting information). In addition, a slower mode appeared, which was assigned to the motion of chain-like clusters (Zinn *et al.*, 2023 ▸).

Fig. 8 ▸(*b*) depicts the corresponding modeling including *S*_ch_(**q**) as given by equation (23) together with equation (18) in equation (1). The doublet configuration is required for reproducing the butterfly wings feature. Then the packing constraints would require at least two intertwined chains so that the caps of doublets are bridged by the neighboring doublet of the other chain, as schematically shown in Fig. 3 ▸. The two uncoated surfaces are expected to be repulsive due to surface charges on the silica core. Otherwise, the suspension would be flocculated before applying the magnetic field. The vertical streak in Fig. 8 ▸(*a*) is nearly reproduced by the simulated pattern in (*b*). The mean linear dimension of the chains is much longer than can be captured by the *q* range probed. Nevertheless, the sharpness of the streak suggests that several tens of particles may be arranged linearly.

For a comparison, Fig. 8 ▸(*c*) demonstrates that when the chains are oriented along the beam direction, the simulated pattern using equations (19) and (24) is isotropic with an increase in intensity at low *q*. An analogous condition was involved in the USAXS measurement presented in Fig. 6 ▸(*b*), where the low *q* excess intensity is shadowed by the beamstop. Fig. 2 of the supporting information compares the vertical and horizontal sections of the scattering pattern with the model for |**B**| = 1.0 mT and 1 T along the *y* axis. The remaining mismatch can be partly attributed to the lower resolution employed for the calculation to achieve a reasonable computing time. Further improvements could be obtained using curved chains. Moreover, the presence of multiple twined chains cannot be excluded.

## 5. Conclusions

The magnetic field induced orientation and assembly of dilute suspensions of silica–nickel Janus particles suspended in water were investigated by means of USAXS. Even at low volume fractions, ϕ ≃ 10^−4^, and low fields, 1.0 mT perpendicular to the X-ray beam direction, particles oriented significantly. The observed anisotropic scattering features were quantified in terms of a semi-analytical model. No alignment was observed when the field direction was turned along the X-ray beam. This behavior was also reproduced by the presented model. In addition, the model quantitatively describes the orientation-averaged isotropic scattering profile over a broad range of *q* yielding critical information about the thickness variation of the cap and its surface coverage. At higher fields, JPs assembled into locally elongated chain-like structures along the field direction, as indicated by the anisotropic scattering feature at very low *q* values, which was replicated by the model.

Furthermore, simultaneous XPCS measurements enabled probing of the ensemble-averaged dynamics of these optically opaque magnetic colloids in an external field. Despite the alignment of the particles, the dynamics remained diffusive but with a progressively smaller diffusion coefficient and nearly isotropic until the formation of chain-like structures (Zinn *et al.*, 2023 ▸). Upon the formation of chain-like structures, the dynamics became anisotropic with a weaker *q* dependence (Fig. 1 of the supporting information).

The description of the orientation behavior of these JPs by a quantitative model opens a new perspective for further investigation of this system. In particular, their orientation dynamics in a time-varying field as well as phoretic dynamics in gradient magnetic fields will be worth exploring. The emergence of magnetic forces affecting the interparticle interactions and dynamics in weaker fields, |**B**| ≤ 0.1 T, is of relevance to biomedical applications. As demonstrated here, USAXS together with ultrasmall-angle XPCS provides a powerful platform for the simultaneous investigation of the ensemble-averaged microstructure and dynamics of anisotropic colloidal systems. The theoretical framework presented here would be useful for the quantitative interpretation of the scattering data from many other oriented systems. 

## Supplementary Material

Supporting information. DOI: 10.1107/S205225252301000X/fs5227sup1.pdf

## Appendix A

Scattering amplitude of the spherical core with radius *R* [volume  ] and constant scattering contrast  (Guinier, 1994 ▸):

and

where *j*_1_(·) is the first-order spherical Bessel function (Abramowitz & Stegun, 1964 ▸).

## Appendix B

Three-dimensional rotation of the vector **r**′:

## Appendix C

The scattering amplitude for the Janus cap is given by

To solve the integral as far as possible analytically, the integration over φ′ can be rewritten as

Using the integral representation of the zeroth-order Bessel function of the first kind (Abramowitz & Stegun, 1964 ▸):

Finally, *A*_cap_(*q*_*y*_, *q*_*z*_, *R*, *d*, γ) can be analytically written as

or in a more reader-friendly way as

## Appendix D

Integral over *r*′:

The integral over ϑ′:

For large values of *M* and *N*, the integral can be approximated and

## Appendix E

Rotations of the position vectors:

and
