# Supplementary material for: Orientational ordering and assembly of silica–nickel Janus particles in a magnetic field
Source: IUCrJ. 2024 Jan 1;11(Pt 1):109–19. doi: 10.1107/S205225252301000X (PMC10833383; doi:10.1107/S205225252301000X)
Supplement: Supplementary file 1 [file m-11-00109-sup1.pdf]

IUCrJ

Volume 11 (2024)

Supporting information for article:

Orientational ordering and assembly of silica–nickel Janus particles in a magnetic field

Gouranga Manna, Thomas Zinn, Lewis Sharpnack and Theyencheri Narayanan

Orientational ordering and assembly of silica-nickel Janus particles in a magnetic field

Gouranga Manna,[a] Thomas Zinn,[a,b] Lewis Sharpnack[a,c] and Theyencheri Narayanan [a]*

[a]*ESRF - The European Synchrotron, 38043 Grenoble, France,* [b]*Present address: Diamond Light Source, Didcot OX11 0DE, United Kingdom, and* [c]*Present address: Department of Earth Science, University of California, Santa Barbara, CA 93106, U.S.A.. E-mail: narayan@esrf.fr*

Supporting Information (SI)

1. XPCS

Figure 1 presents a set of normalized intensity-intensity autocorrelation functions, $g_2(q,t)$, measured at two different fields (a) 0.1 mT and (b) 1.0 T for a JPs suspension with $\phi \simeq 1\times10^{-4}$. The top and bottom rows display $g_2(q,t)$ along the y and z direction, respectively, and $\vec{B}$ was along y-axis. The dynamics is clearly isotropic at 0.1 mT and the $g_2(q,t)$ could be described by a single-exponential decay as indicated by the solid lines (Zinn *et al.*, 2023). The relaxation rates, $\Gamma_e(q)$, shown in Figure 1(c) obtained from the fits display a q^2-dependence as expected for free translational diffusion. The deduced value of diffusion coefficient, $D_0 = 0.81\,\mu m^2 s^{-1}$ corresponds to an apparent hydrodynamic radius, $R_H = 281$ nm, assuming solvent viscosity, $\eta_s = 0.95$ mPas and a temperature of 295 K. A comparison of the $g_2(q,t)$ at 1.0 T in Figure 1(b) reveals that

the decay of $g_2(q,t)$ along z direction can still be described by a single-exponential function. However, along $\vec{B}$ direction, $g_2(q,t)$ decay more like a compressed exponential function with an exponent close to 1.4 and the corresponding $\Gamma_e(q)$ manifest a weaker q-dependence signifying an anomalous dynamics.

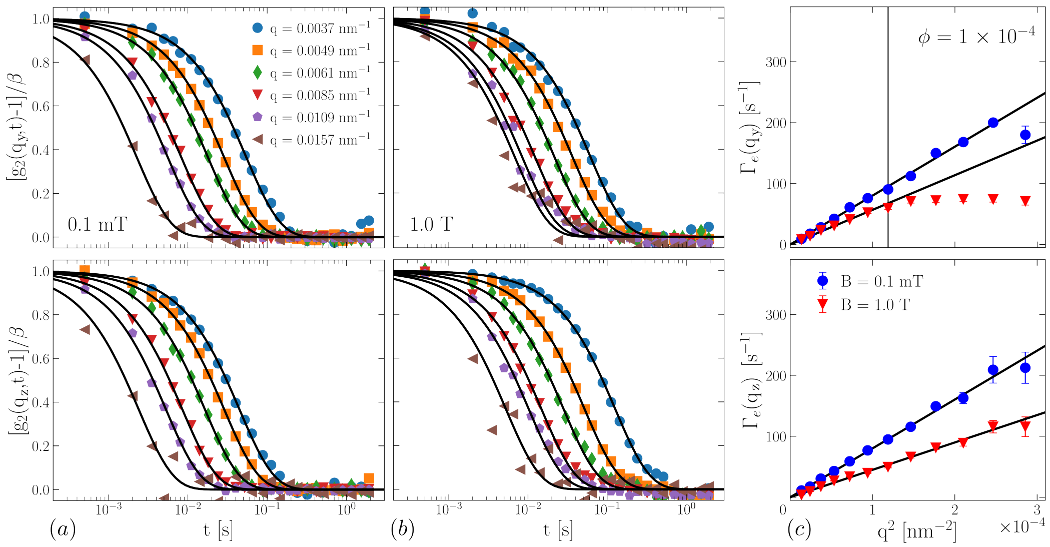

Fig. 1. Normalized intensity-intensity autocorrelation functions, $g_2(q,t)$, from JPs ($\phi \simeq 1 \times 10^{-4}$) measured at two different values of $|\vec{B}|$ (a) 0.1 mT, (b) 1 T for a range of q-values along y and z directions, respectively. The horizontal and vertical sections were taken over an azimuthal range of $\pm$ 5°. Corresponding dispersion relationship is shown in (c) for both $\vec{B}$.

2. USAXS modeling

A comparison of the 1D USAXS profiles for the data shown in Figures 5(a) and 8(a) in the main text.

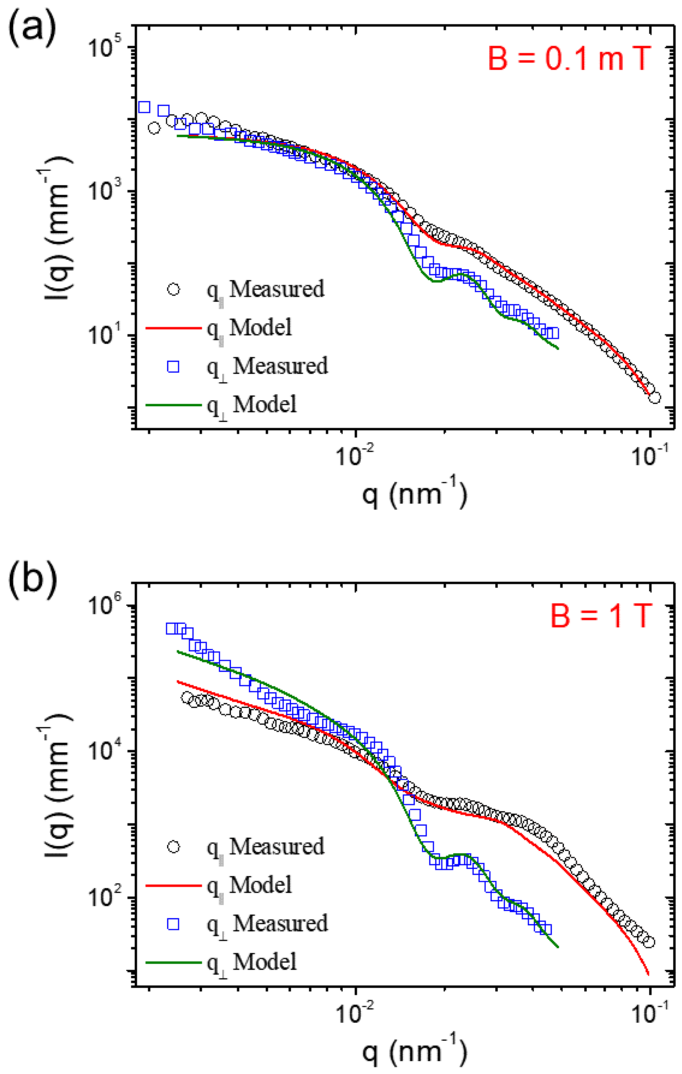

Fig. 2. Comparison between the experimental and model USAXS profiles at two different $\vec{B}$ values (a) 0.1 mT and (b) 1 T perpendicular to the X-ray beam direction (y-axis). The vertical and horizontal sections were taken over an azimuthal range of ± 5°.

References

Zinn, T., Sharpnack, L. & Narayanan, T. (2023). *Soft Matter*, **19**(13), 2311–2318.
